# Characterization of Bronze Surface Layer Formed by Microarc Oxidation Process in 12-Tungstophosphoric Acid

**DOI:** 10.3390/ma3010110

**Published:** 2009-12-31

**Authors:** Ubavka B. Mioč, Stevan Stojadinović, Zoran Nedić

**Affiliations:** 1 Faculty of Physical Chemistry, University of Belgrade, P.O. Box 47, 11158 Belgrade 118, PAC 105305, Serbia; E-Mail: zoran@ffh.bg.ac.rs (Z.N.); 2 Faculty of Physics, University of Belgrade, Studentski trg 12-16, 11000 Belgrade, Serbia; E-Mail: sstevan@ff.bg.ac.rs (S.S)

**Keywords:** 12-tungstophosphoric acid, microarc oxidation, tungsten bronzes

## Abstract

This paper is a brief review of our recent research into novel uses for heteropoly compounds as precursors for thin films that can be used as catalysts and materials with good optical, conductive and other characteristics. In view of this, we have chosen thin film obtained with 12-tungsphosphoric acid on aluminum substrates. In all cases, a relatively new, microarc oxidation technique has been used to prepare oxide coatings on substrate surfaces. Advanced physicochemical methods, AFM and SEM-EDS, XRD, Raman and Micro-Raman, and luminescence spectroscopy, as the most powerful techniques, have been used for the characterization of new materials. Possible applications have been discussed as well.

## 1. Introduction

Polyoxometalates (POMs), although having more than a hundred years of history, continue to attract the attention of researchers as catalysts, solid superionic proton conductors at room temperatures, applicable in different electrochemical devices, and also as photochromic, biochemical and biomedical active materials [1,2,3,4,5,6,7]. The best-known group of polyoxometalates (POMs) are the heteropoly compounds (HPCs) with the Keggin anion structure. The use of HPCs as solid super ionic proton electrolytes started with the study of Nakamura *et al.* [3], who have found that heteropoly acids (HPAs), at room temperature, have proton conductivity between (100–1) × 10^-3^ S/cm. 12-Tungstophosphoric (WPA), 12-molybdophosphoric (MoPA) and 12-tungstosilicic acid (WSiA) are the three best-known acids with Keggin structures [1,2,3,4]. Acids, neutral and acidic salts of alkali and alkaline-earth salts of HPAs, as well as bronzes [4,8,9,10,11], obtained from cited compounds as precursors, have been also the subject of our continuing and systematic investigations.

Systematic experimental investigation of HPAs, particularly their structure and activity, have led to the conclusion that most of their characteristics are governed by the dynamic equilibrium of proton and protonic entities as well as the specific surface area, depending on particle size [4,12,13]. Primary and secondary structures of HPCs define their high redox properties, polarity, surface charge distribution, electron and proton transfer/storage ability, high charges and high ionic weight of heteropoly anions, and explain their applications. Drawbacks of these compounds are their sensitivity to environmental conditions, temperature and relative humidity. These disadvantages can be overcome by incorporation of the acid into various stable porous supports and formation of different composites [14,15,16,17] or by neutralization of the acid with large cations (K^+^, Rb^+^, Cs^+^, NH_4_^+^) to form porous insoluble salts, which are less sensitive to the ambient conditions [18,19,20,21,22]. All these compounds exhibit high proton conductivity and can be used as solid electrolytes in fuel cells, or as catalysts due to their high specific surface areas.

Now, in the time of economical crisis and fast climatic changes, rechargeable solid-state batteries and fuel cells have become a necessity and worldwide efforts are presently underway to develop new materials for rechargeable solid-state energy sources. Fast proton conductors have their own important role in solving this problem [23,24,25,26].

In this paper, special attention is paid to the coating of phosphate tungsten bronzes, obtained with WPA as precursor, on aluminum. The coating has unique strength, as well as catalytic and optical characteristics and as such can be used in many fields, such as catalysis, aerospace technology, microelectronics, *etc.* [27,28,29,30]. A relatively novel technique, microarc oxidation (MAO), has been used to prepare oxide coatings on support surfaces [29]. In many cases, HPCs have been used because of the low temperature of their structural phase transitions to bronzes, at about 600 °C and lower. At structural phase transition temperatures, the investigated tungsten and molybdenum bronzes, of ReO_3_ type structure, are stable inorganic solids and can have different general formulas [31,32]. These compounds have been studied due to their photoelectrochromic [33], magnetic [34], and other properties [35,36,37,38,39,40]. A common structural feature of these materials is the MO_6_ (M = W, Mo, V, Nb) octahedral unit, which is repeated along three directions through shared corners [37]. This structural arrangement provides empty channels, where monovalent ions such as Li, Na, K, Cs, H^+^ and proton entities can be inserted to stabilize the structure and to form a great variety of compounds.

Metal-phosphate tungsten bronzes (M_x_-PWB; 1≤ x ≤3), with different structural arrangements can be produced if WO_6_ units are replaced with mono-phosphate (PO_4_) or di-phosphate (P_2_O_7_) groups [39,40]. The main families of monophosphate tungsten bronzes A_x_(PO_2_)_4_(WO_3_)_2m_ could be either with pentagonal (MPWB_p_) or with hexagonal tunnels (MPWB_h_), and diphosphaphate tungsten bronzes A_x_(PO_4_)_2_(WO_3_)_2m_ mainly have hexagonal tunnels (DPWB_h_). Two model structures with (m = 4 and m = 10), also exist and are solved. A_x_ is an inserted cation, usually Li and Na for monophosphate bronzes and larger ones K, Cs Rb and Ba for diphosphate bronzes. Their structure may be describe as building of polyhedra sharing oxygen corners made of (WO_3_)-type slabs joined by silices of phosphate PO_4_ or diphosphate groups or P_2_O_7_. WO_6_ octahedra could be find inside a slab of WO_3_ [37].

The first methods for metal phosphate tungsten (M_x_-PWB) or molybdenum bronze (M_x_-PMoB) production were thermal ones, based on solid state processes, under extremely crude conditions: high-temperature (≈1,000 °C) and high-pressure. They were based on heating of powdered metal oxide mixtures, M_x_O_z_, or Mo or W and P_2_O_5_ [33,34,35,36,37,38]. The processes were too long and the homogeneity of obtained bronzes was questionable.

Recently, a method of electrochemical insertion of electropositive metal ions, of appropriate size, into phosphate tungsten bronzes have been developed (Li_x_P_8_W_12_O_52_, Li_x_P_4_W_12_O_42_ and Li_x_P_4_W_8_O_32_) [39,40]. In this way, a wide variety of different phosphate tungsten bronzes have been obtained: perovskite type bronzes formed with small cations such as Li and Na, tetragonal tungsten bronzes with Na and K, and hexagonal ones formed with larger cations such as K, Rb and Cs. The obtained bronzes have been found to be good metallic conductors, because metal filled the W-O-W hybridized π* conducting band.

Phosphate bronzes have been also obtained in the process of thermal treatment of HPCs, after exothermic phase transition, followed by solid-solid recrystallization of Keggin`s anion [4,41,42,43]. Metal-doped bronzes (Mx-PWB) can be obtained if the precursors are HPA salts.

We have also developed an ultrasonic spray pyrolysis (USP) method to obtain fine, spherical, nanostructured PWB particles starting with WPA, or metal-WPA salt solutions [44,45,46]. This method enables one to control and maintain a uniform chemical and phase composition of final powder products and, simultaneously, to produce micron and submicron size particles. The agreement between experimentally obtained and theoretically predicted data of particle sizes proved good, and showed that 60% of particles had the size between 30 and 50 nm [45,46].

As coating supports, a variety of oxides such as Al_2_O_3_, TiO_2_, Ta_2_O_5_, NiO and Nb_2_O_5_ have been used [29,30,46,47]. Few authors have used HPAs as precursors [48,49] to obtain coatings on support surfaces. Based on our previous knowledge and experience, we have made an effort to improve the film characteristics for their better commercial exploitation, by using WPA as precursor.

## 2. Experimental

Many characteristics of the coating depend on sample preparation, Al/Al_2_O_3_, Ti/TiO_2_, WO_x_/ZrO_2_ supports, and also on the characteristics of electrolytes used for anodization of samples.

### 2.1. Sample Preparation

Metal/metal oxide (M/M_x_O_z_) supports can be prepared by a variety of routes. In our case an Al/Al_2_O_3_ support was used. In the experiment, anodic oxide films were formed on high purity (99.999% Goodfellow) cold-rolled aluminum samples of 30 mm × 10 mm × 0.25 mm dimensions. The anodic oxidation process was carried out in an electrolytic cell with flat glass windows. Platinum wires were used as cathodes. During anodization, the electrolyte circulated through the chamber–reservoir system, and the control temperature sensor was situated immediately by the sample. The temperature of the electrolyte was maintained to within 0.1 °C during anodization. Before anodization, the aluminum was degreased in acetone using an ultrasonic cleaner and then annealed for 4 h at 450 °C to remove mechanical stresses and recrystallize the aluminum. The surface of aluminum samples was electropolished in a mixture of perchloric acid and ethanol (1:4 in volume) under a constant voltage of 18 V for 1 min below 5 °C, to obtain smooth-surface samples for anodization. After electropolishing, the samples were rinsed with ethanol and dried. For the production anodic films, electrolyte solution of WPA, concentration of 10^-3^ mol/dm^3^ was used. In all cases, the solution pH was controlled to be pH = 1, where the Keggin structures of HPCs are stable in aqueous solutions [50].

### 2.2. Experimental Techniques

*Atomic Force Microscopy (AFM):* A Veeco Instruments model Multimode V was used to analyze the morphology of anodized aluminum samples and oxide coating surfaces, with tapping mode in air.

*Scanning Electron Microscopy (SEM):* The images of film samples were examined by means of a Joel 840A instrument equipped with EDS analyzer. SEM-EDS analysis was also used to characterize the chemical composition of formed oxide coatings.

*X-Ray Diffraction (XRD):* The crystallinity of samples was analyzed by XRD, using a Phillips PW 1050 instrument, with Cu K_α1,2_ radiation. Diffraction data were acquired over scattering angle 2θ from 10 to 90° with a step of 0.050° and acquisition time of 1 s/step.

*Spectroscopic analysis:* The Raman and Micro Raman spectra of polycrystalline samples were recorded on a Thermo DXR Raman microscope, using the 532 nm laser excitation line and constant power of 10 mW. The resolution of all measured spectra was 4 cm^-1^. Micro-Raman mapping was carried out under the following conditions: exposure time 5.00 s, number of exposures 10, number of background exposures: 512. Spectral luminescence measurements under breakdown conditions (MAO) were performed utilizing a spectrograph system based on Intensified Charge Coupled Device (ICCD) camera intended for time-resolved measuring of very weak light intensity in a wide range of wavelengths [51]. Optical detection system consisted of a large–aperture achromatic lens, a Hilger spectrograph with diffraction grating 1,200 grooves/mm (wavelength range of 43 nm) and a very sensitive PI–MAX ICCD cooled camera with high quantum efficiency manufactured by Princeton Instruments. To reduce the dark current the CCD chip is cooled at – 40 °C using Peltier devices. The system was used at several grating position with overlapping wavelength range of 10 nm.

## 3. Results and Discussion

### 3.1. Coating Formation by MAO Process

Microarc oxidation (MAO) is an advanced high-voltage anodizing process of plasma-assisted electrochemical conversion of a metal surface to oxide coatings. The process is known to involve a large number of short-lived sparks (electrical discharges), caused by localized electrical breakdown of the growing coatings. Micro discharge characteristics determine the thermal and chemical conditions on the oxidizing surface, and play an important role in the formation of phases, structure and stress state of the coatings.

In accordance with the dynamics of the discharge, the process occurs in two steps, *i.e.*, ionization and condensation. In the first step, impact or thermal ionization occurs in the discharge area. The processes occurring here are primarily compound dissociations on protons and/or proton entities and Keggin’s anions. The plasma in the discharge channel reaches high temperatures and pressures for a very short time shorter than 10^−6^ s [52]. The electric field in the discharge channel separates charged particles in plasma. Positive ions pass into the electrolyte while negative ions participate in processes occurring on the electrode surface. During the second step, the temperature rapidly drops and the components of the plasma form products that are condensed within the discharge channel.

### 3.2. Microscopic Analysis: AFM and SEM-EDS

**Figure 1:**
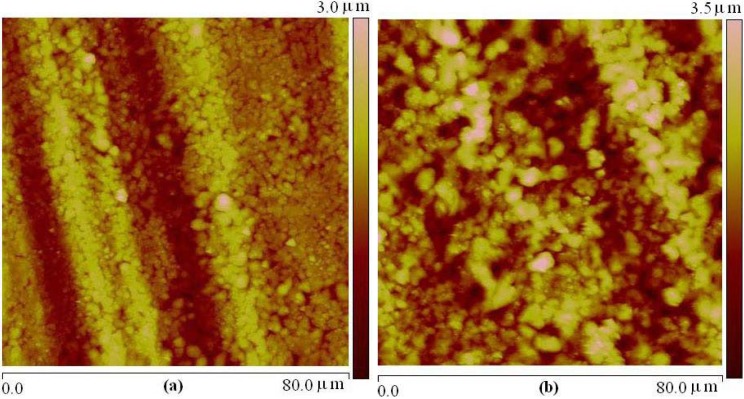

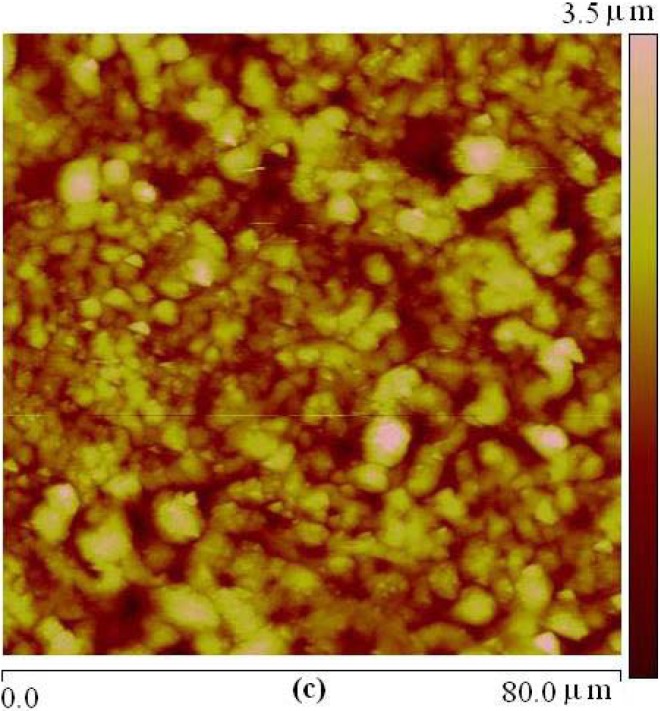
AFM surface morphology of microarc oxidation coating formed in different treatment period: (a) 5 min; (b) 15 min; (c) 30min.

The AFM images have been used for surface morphology investigation. The morphology of oxide coating surfaces on aluminum anodized for 5, 15 and 30 min is shown in Figure 1. The surface is characterized by micropores of different sizes, between 0.9 to 1.6 μm, and shapes. Film and/or islands of tungsten compounds are evident on the metal support. These islands are of different sizes, between 500 to 1,300 nm, with evident substructure. Islands have also different colors, black and green, which could be evidence of several tungsten compounds formed. Green color is a proof that phosphate bronzes were formed. The evolution of surface morphology is clearly demonstrated by a visible decrease in micropores number and increase in micropores size with prolonged treatment time.

**Figure 2:**
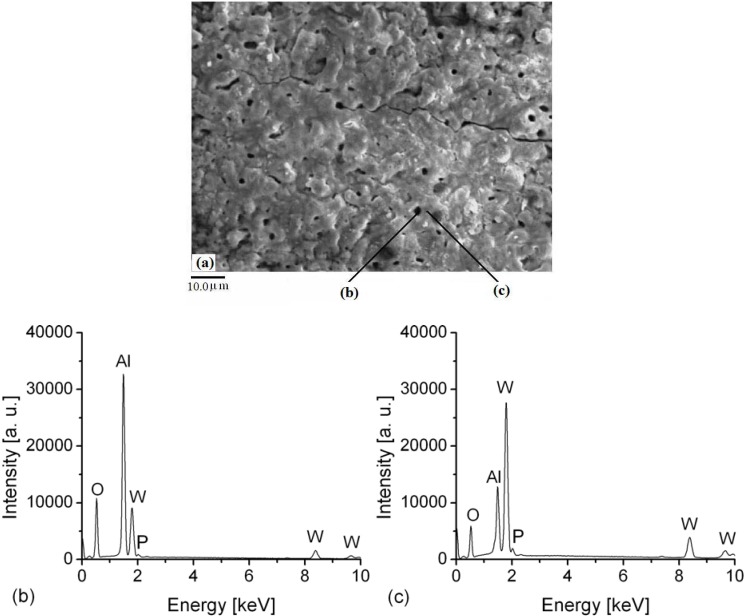
(a) SEM image of microarc oxidation coating treated for 30 min; (b) EDS spectrum recorded in discharge channel; (c) EDS spectrum recorded around discharge channel.

The coating surface was also investigated by SEM and EDS. The EDS results revealed the element distribution in and around the discharged channel, as shown in Figure 2. It is clear that the Al content is much higher in the discharged channel than in the particle (Figure 2b), while the particles are richer in tungsten than the discharged channel (Figure 2c).

### 3.3. Micro-Raman Spectroscopy

**Figure 3:**
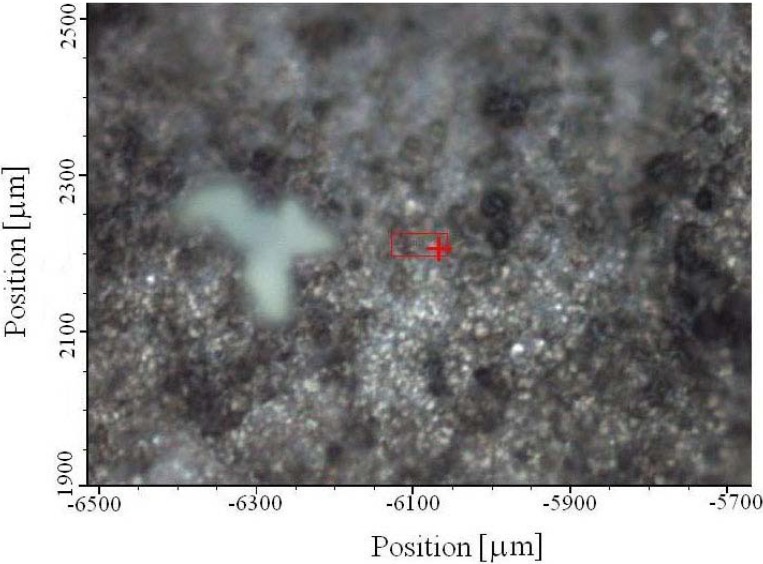
Micro-Raman image of oxidation coating with mapping area.

Micro-Raman spectroscopy is a powerful method in the study of surface chemical bonding. With modern instruments, where the mapping is possible, two-dimensional or three-dimensional distribution of chemical species can be obtained. It means that it is possible to get chemical composition of each island, or cluster as evident in AFM image, Figure 1. Among series of islands, some green ones are clearly seen, Figure 3.

**Figure 4:**
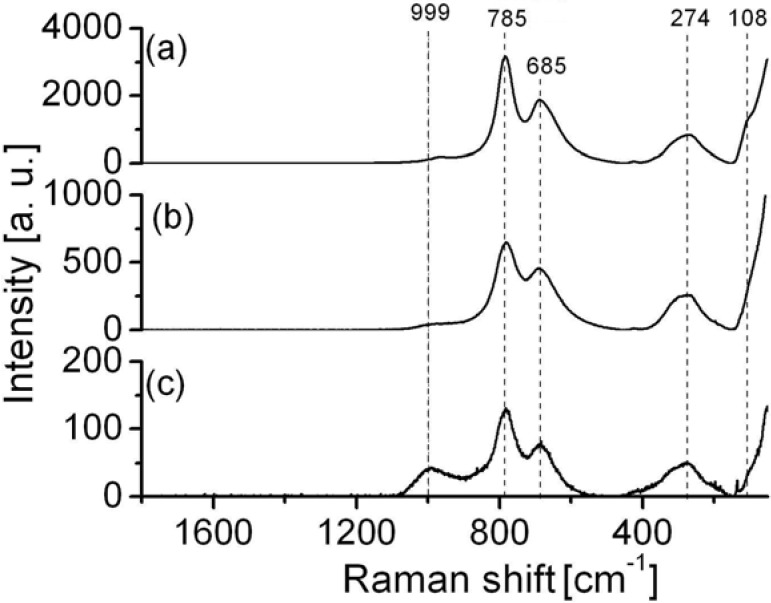
Raman spectra of phosphate tungsten bronzes: (a) reference spectrum; (b) spectrum taken from the middle of green spot; (c) spectrum taken on edge of green spot from Figure 3.

Based on our previous experience gained in studying phosphate bronzes, green color is characteristic of phosphate tungsten bronzes (PWB), obtained in the process of thermal treatment of WPA [4,42,43,44,45,46] . A reference Raman spectrum of PWB is given in Figure 4a. In the same figure Raman spectra taken in the middle of green spot (b) and on its end (c) are given, too. The spectra are identical and the band at about 1,000 cm^-1^, characteristic of phosphate group ν_1_ (1,035 cm^-1^) is relatively broad because of possible overlapping with the band of ν_3_ vibration at 1,075 cm^-1^. Bands at about 785, 685, 274 and 108 cm^-1^ ascribed to WO vibrations from bronzes are also evident, Figure 4. It means that the composition of the compounds obtained by microarc discharge on Al_2_O_3_ support, from aqueous solution of WPA, are identical to those obtained by thermal treatment of WPA as precursor [4].

On the basis of Raman spectra and characteristic bands, it is difficult to speak of the form and structure of tungsten oxide, although they are very close to the ReO_3_ type of bronzes with cubic structure, characterized by bands at about 805 and 706 cm^-1^, assigned to W-O-W stretching, and at 273 cm^-1^, assigned to W-O-W bending vibrations [53,54]. Some other processes can also influence the position of bands in Raman spectra: surface tungsten oxides on aluminum can form a complex with alumina that can distort the octahedral and/or the tetrahedral structure of tungsten oxides on the surface and provoke appearance of W=O double bond, too. In any case, it is necessary to pay more attention to the structure of tungsten oxides on alumina surface.

**Figure 5:**
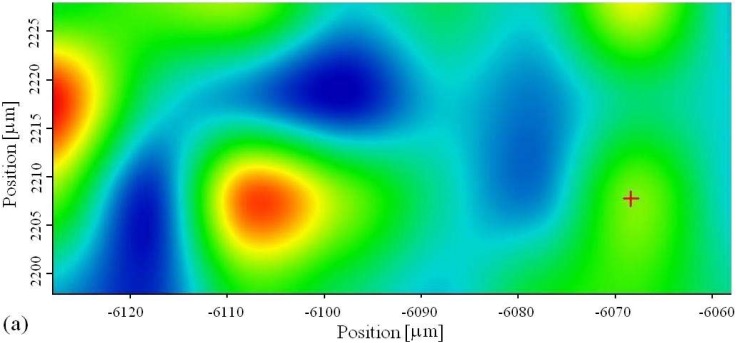

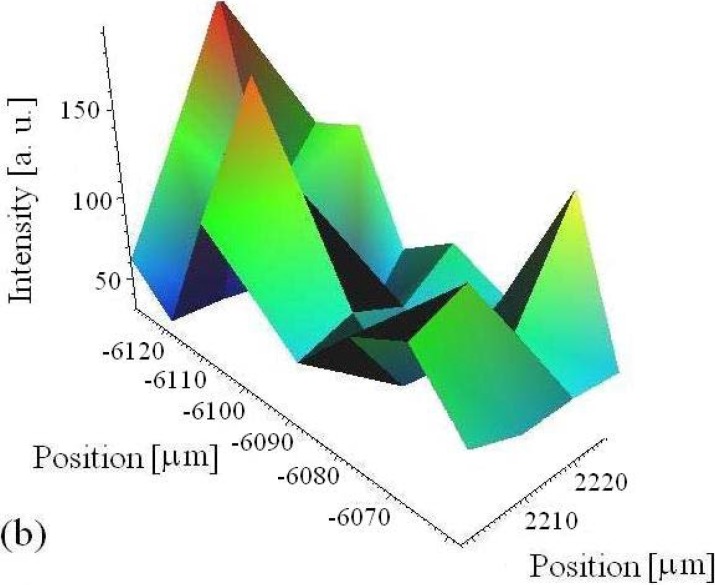
Mapping image of phosphate from tungsten bronze in: (a) 2D; (b) 3D.

Mapping has been done out of green zone, to see whether there are some small islands of phosphate tungsten bronzes. The place where it has been done is marked as a rectangle in Figure 3. The mapping has been done first for phosphate group at 994 cm^-1^, Figure 4, and the results are given in 2D and 3D coordinates, Figure 5.

**Figure 6:**
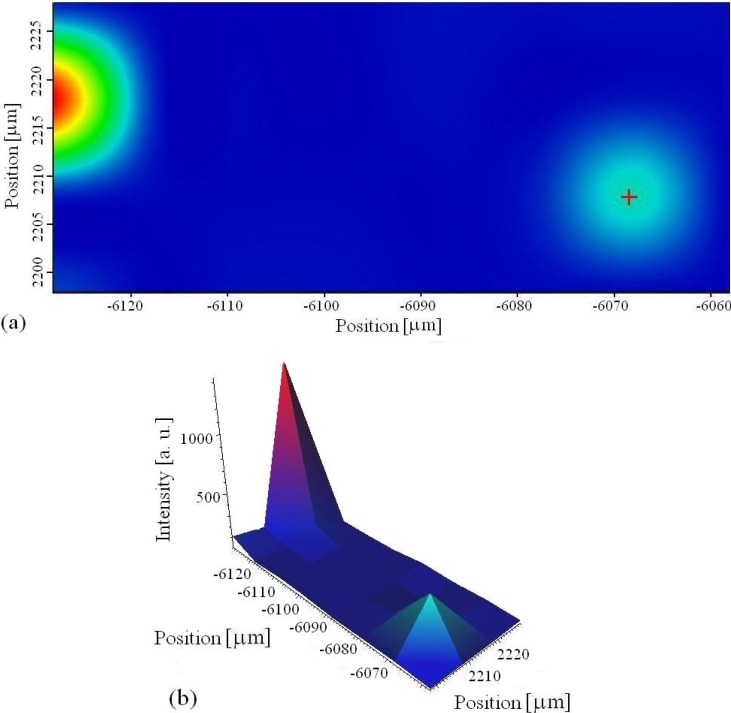
Mapping image of tungstant oxides from bronze in: (a) 2D; (b) 3D.

The coating mapping procedure, for tungsten, has been done at the 777 cm^-1^ line, Figure 6. The presence of W-oxides is also confirmed. The presence of pure WO_3_ however cannot be excluded, especially in microarc discharged places, where the temperatures are the highest.

Phosphate tungsten bronzes [4,42,43,44], obtained on the substrate, have a conductivity of 7 × 10^-4^ S/cm at room temperature, measured at four points. Compared with that for thermally obtained bronzes (σ ≈ 2 × 10^-5^ S/cm), the conductivity is more than one order of magnitude higher, which can be explained by the higher specific surface area of the coating than that of thermally obtained PWB. The conductivity of bronzes increases with increasing temperature, and at 200 °C, AC conductivity is 1.9 × 10^-3^ S/cm. So, the bronzes could be used as solid electrolytes in the medium temperature range, above 100 °C [45].

Another important characteristic of heteropoly compounds is their high catalytic activity. Being soluble in polar solvents and stable in solid state they can be used in either homogenous or heterogeneous catalytic processes. They are very strong Brönsted acids and efficient oxidizing agents. HPAs are known as super acids with the strongest acid sites among inorganic acids, even better than sulfuric acid [1,2,55,56,57]. Their salts are often used as catalysts also, especially because of their high specific surface areas. Cesium salt of WPA (Cs_2.5_H_0.5_PW_12_O_40_) is commonly accepted to be the most active solid catalyst in many reactions, for example for methanolysis of triglicerides [22].

Even coatings obtained from HPCs show interesting catalytic behavior and have attracted significant attention. HPAs on zirconia have a mesoporous structure, and higher specific surface area than acids themselves. Metal cations or metal oxide clusters interact with oxide supports and form structures that stabilize the protons responsible for Brönsted acidity. This process is much easer in the case of tungsten or molybdenum, as atoms with mixed valence and reversible process of oxidation↔reduction. WO_3_/ZrO_2_ appears to contain the strongest acid sites among tungsten oxide based materials reported in literature. Therefore, such catalysts are very frequently used in n-pentane isomerzation at low temperatures, butene dimerization and others reactions [27].

### 3.4. XRD Analysis

**Figure 7:**
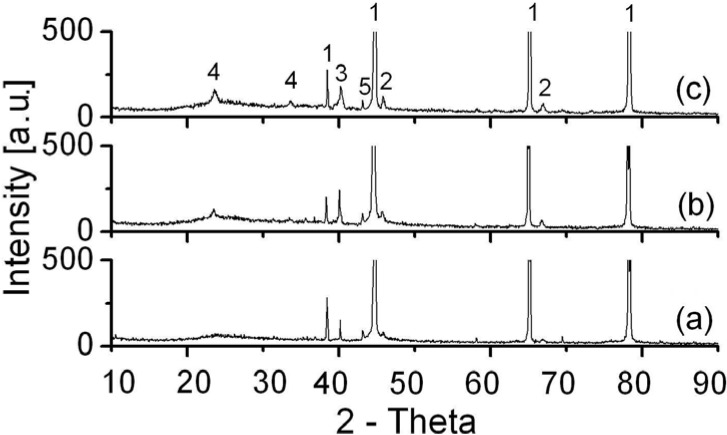
XRD spectra of microarc oxidation coating formed in different treatment period: (a) 5 min; (b) 15 min; (c) 30min. Designation of reflections: Al-(1), γ-Al_2_O_3_-(2), W-(3), WO_3_-(4), α-Al_2_O_3_-(5).

Figure 7 shows XRD spectra of the surfaces of coatings obtained by MAO in different treatment periods. The coating is partly crystallized and mainly composed of γ-Al_2_O_3_, α-Al_2_O_3_ and WO_3_. Elemental Al mainly originates from the support and therefore the Al diffraction lines are so strong. Good agreement between the results of XRD and those obtained by other experimental methods, used in investigation of coating, has been obtained.

### 3.5. Coating Optical Emission

**Figure 8:**
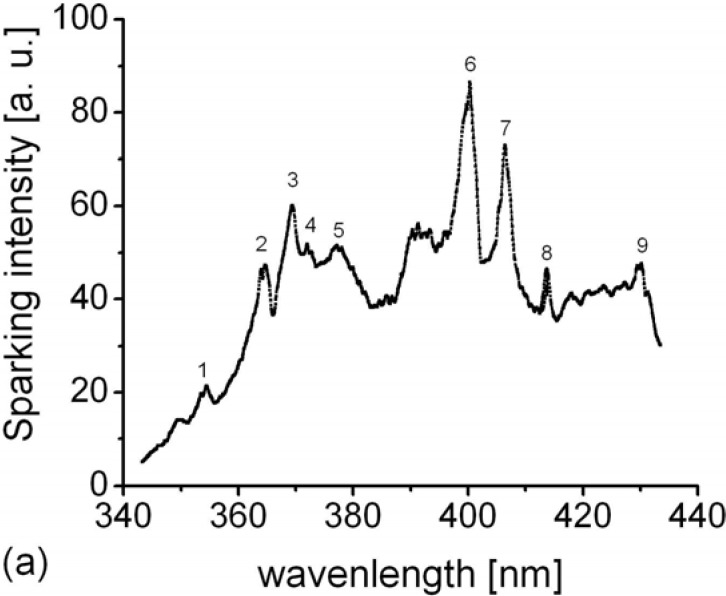

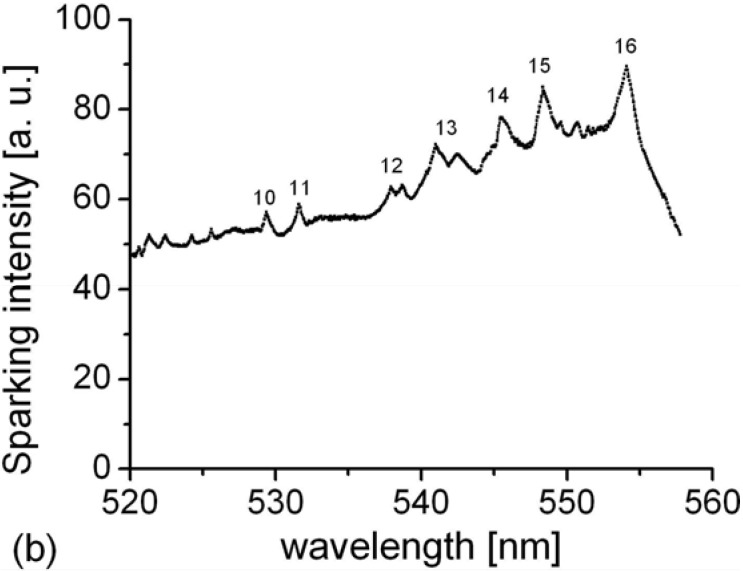
Luminescence spectra recorded during MAO in the spectral range: (a) 340 nm – 440 nm; (b) 520 nm – 560 nm.

Optical emission spectroscopy has been used, for the *in-situ* monitoring of the light emission in order to understand the role of plasma in coating formation. Typical spectra obtained during anodization of aluminum in WPA, in the ranges from 340 to 440 nm and from 520 to 560 nm, are shown in Figure 8.

Intensive emission spectra are evident. Cleary extended lines indicate atomic origin. We used NIST atomic spectra database in order to assign each line to the characteristic atomic transition [58]. The results are listed in Table 1. By inspection, we found that only spectral systems that could contribute to our spectrum recorded in the range from 340 to 440 nm were transitions in W I and W II. The transitions of phosphorus (P II) are responsible for the appearance of the observed emission spectra in the range from 520 to 560 nm. Moreover, we detected several intensive emission lines in the range from 390 to 720 nm arising from transitions in atoms Al, O, and H, also detected under breakdown conditions during anodizing of aluminum in some other electrolytes (boric acid + borax, ammonium tartrate, *etc*.) [52].

As anodization has been performed in an electrolytic solution of heteropoly acid W I and W II and P II lines in spectra are expected. At the same time, this finding can confirm the existence of excitation of coating-phosphate tungsten bronzes and/or WPA from the solution. As in plasma, the processes are rather complicated and dependent on temperature, and some other chemical species can be formed such as WO_3_, or some other oxide forms depending on the substrate and/or electrolyte chemical composition.

All our experimental results, obtained by SEM-EDS, XRD and optical emission spectroscopy, confirm that the coating is mainly formed of phosphate tungsten bronzes contrary to other previous literature data. Even Lukiyanchuk *et al.* [48] have found that the coating, from electrolyte solutions with iso- and hetropoly compounds, is formed of WO_3_. Usually, the researchers do not find phosphorous in the coating layer. In recent papers [59,60], the possibility of forming coatings of different forms of compounds containing phosphorous and/or some forms of POMs has been indicated. Mane *et al.* [49] reported on the formation of a salt layer Tl_3_PMo_12_O_40_ stable up to 286 °C. It is evident that the problems related to thermolysis of HPCs have not been solved and that further systematic investigations are necessary.

**Table 1:** Wavelength, transition strength, and relative intensity of atomic and ionic species.

	Ion	Observed Wavelength Air [nm]	Relative Intensity	*A_ki_* [s^-1^]	*E_i_* [cm ^-1^]	*E_k_* [cm ^-1^]
1	W I	353.5539	100	7.6e+06	13307.10	41583.20
	W I	354.5220	200	3.2e+06		28198.90
2	W II	364.1407	113	9.91e+06	8711.274	36165.356
	W II	364.5595	134	1.46e+06	14967.745	42390.287
3	W I	370.7922	300	2.9e+06	2951.29	29.912.85
4	W I	372.2235	60	1.19e+07	19648.54	46506.37
5	W I	376.8447	250	3.47e+06	1670.29	28198.90
	W I	378.0772	200	4.2e+06	2951.29	29393.40
6	W I	400.8753	1000	1.63e+07	2951.29	27889.68
7	W I	407.4358	600	1.0e+07	2951.29	27488.11
8	W I	413.7457	80	8.4e+06	3325.53	27.488.11
9	W I	429.4606	800	1.24e+07	2951.29	26229.77
10	P II	519.613	400	5.5e+07	87124.60	106001.25
11	P II	531.607	250	2.4e+07	86743.96	105549.67
12	P II	537.820	250	1.1e+07	105302.37	123890.81
	P II	538.688	300	2.3e+07	86743.96	105302.37
13	P II	540.972	200	9.3e+07	86743.96	105224.06
	P II	542.591	400	6.9e+07	87124.60	105549.67
14	P II	545.074	400	3.3e+07	105549.67	123890.81
15	P II	548.355	200	1.5e+07	105224.06	123455.46
16	P II	554.114	200	4.5e+07	105302.37	123344.19

## 4. Conclusions

In this paper results of coating formation on Al/Al_2_O_3_ substrate, during anodization of aqueous solution of WPA are presented and discussed. On the base of experimental results it has been concluded that compositions of coating, obtained by thermolysis of WPA as precursor, is identical with phosphate tungsten bronzes obtained during thermal treatment of solid WPA. The results of all used experimental techniques, presented in this paper, confirm that in the process of microarc oxidation of formed coating is phosphate tungsten bronze. Method of microarc oxidation of HPAs on different substrates could be taken as one new method for phosphate tungsten bronze obtaining.

Discussed process of thermolysis of HPAs is influenced by many factors, composition of electrolytes, preparation conditions and the others. Composition and structure of coating defiantly require forward systematical investigations.
